# Novel Approaches for the Delivery of Anti-HIV Drugs—What Is New?

**DOI:** 10.3390/pharmaceutics11110554

**Published:** 2019-10-28

**Authors:** José das Neves

**Affiliations:** 1 i3S—Instituto de Investigação e Inovação em Saúde, Universidade do Porto, 4200-135 Porto, Portugal; j.dasneves@ineb.up.pt; Tel.: +351-220-408-800; 2 INEB—Instituto de Engenharia Biomédica, Universidade do Porto, 4200-135 Porto, Portugal; 3 CESPU, Instituto de Investigação e Formação Avançada em Ciências e Tecnologias da Saúde, 4585-116 Gandra, Portugal

HIV/AIDS continues to be one of the most challenging individual and public health concerns of our days. According to the latest UNAIDS data, in 2018, roughly 37.9 million individuals were infected with HIV globally, while around 770,000 people died of AIDS-related illness [1]. During that same year, an estimated 1.7 million new infections occurred, mainly due to unprotected sexual intercourse. Investment in the field has been considerable, but a cure to the infection remains elusive. Nonetheless, tremendous advances have been made over the last 36 years since HIV-1 was identified, namely in prevention, diagnostics, and treatment. The development of antiretroviral drugs and the introduction of highly active antiretroviral therapy (HAART) in the mid-1990s—currently referred to as combination antiretroviral therapy (cART)—led to a dramatic shift of AIDS from a fatal disease into a chronic and often stable medical condition [2]. In fact, cART contributed decisively to a steady decrease in the number of HIV-related deaths since the first years of the new millennium [3]. Antiretroviral drugs have also been found useful in the prevention field, particularly in post-exposure prophylaxis or mother-to-child transmission. Treatment as prevention and pre-exposure prophylaxis (PrEP) have further contributed to the reduction of sexually transmitted HIV infections. Long-lasting injectable products and antiretroviral-based microbicides that are currently in late stages of clinical development or regulatory approval may soon provide new options for prevention [4]. Gene therapy and the use of broadly neutralizing antibodies are also attracting a great deal of interest as possible approaches to HIV/AIDS management [5,6,7].

Still, different challenges remain in anti-HIV drug therapy/prophylaxis, and these include the following, among others: (i) the onset of severe adverse effects leading to the discontinuation or interruption of therapy or even prophylaxis [8,9]; (ii) sub-optimal biodistribution and pharmacokinetics, particularly in reservoir sites or mucosae involved in sexual transmission [10,11]; (iii) the occurrence of viral resistance [12]; (iv) troublesome regimens and/or drug delivery routes that lead to poor adherence by patients/users [13,14]; (v) low stability and reduced shelf-life of active molecules, which may be particularly challenging in tropical climates and low-resource regions lacking adequate refrigerated distribution channels and storage [15]; (vi) lack of suitable dosage forms for particular populations (e.g., children and women) [16,17]; (vii) costly drug products that are often inaccessible to populations in need of therapy/prophylaxis [15]; and (viii) social and legal constraints resulting in poor access to and the discontinuation of anti-HIV therapy/prophylaxis [18,19]. The response from the scientific community could not be more affirmative, and novel ideas and concepts have been emerging throughout the last decade or so. More important, innovative products are now under development, holding great promise for mitigating many of the challenges identified above.

This Special Issue presents an exciting series of reviews and original research articles from eminent scientists in academia and different nonprofit organizations involved in the development of antiretroviral drug products, focusing mainly on novel strategies for the formulation and delivery of anti-HIV compounds. Innovative approaches towards improved gene therapy and immunotherapy are also addressed. The presented reports provide not only interesting overviews and opinion on recent developments in the broad field of antiretroviral therapy/prophylaxis and drug delivery, but also describe the development of new products that are currently tracked for clinical testing.

The Special Issue starts with an interesting review by Tsukamoto at Kindai University, Japan, on strategies explored for curing HIV infection using a combination of gene therapy and host immunization [20]. In particular, the author emphasizes the possible role of anti-HIV intracellular immunization using gene silencing, among other approaches, in the protection of bone marrow hematopoietic stem/progenitor cells. Still in the same field, Düzgüneş and Konopka at the University of the Pacific, USA, contributed a stimulating review on a potential strategy for the eradication of cellular reservoirs of HIV [21]. This thought-provoking piece explores how such an objective could be achieved by using suicide gene therapy for killing HIV-infected cells, excision of chromosome-integrated viral DNA, and cytotoxic liposomes targeted to latency-reversed HIV-infected cells.

In the first original research study included in the Special Issue, the group of Veiga at the Complutense University of Madrid, Spain, provides details on the development of mucoadhesive tablets for the vaginal delivery of tenofovir, in the context of topical PrEP [22]. The combination of drug-loaded hydrophobic granules obtained by hot-melt granulation and hydrophilic matrices not only allowed the adhesive behavior of tablets to be increased, but also provided sustained drug release. This new formulation could be potentially beneficial in providing longer protective time windows against male-to-female transmission of HIV. The Special Issue continues with a review article on topical nano-microbicides, this time from my research team [23]. We provide an overview on useful vaginal and rectal platforms for the delivery of anti-HIV microbicide nanosystems. Critical topics and relevant studies concerning the development and testing of vehicles such as aqueous suspensions, gels, thermosensitive systems, films and fiber mats, among others, are detailed. Steinbach-Rankins and colleagues at the University of Louisville, USA, contributed an excellent revision of their own work, as well as the work of others, concerning the development and potential of electrospun fibers for vaginal drug delivery [24]. They particularly focus on the formulation of anti-HIV compounds, and how suitable material selection and the engineering of fibers can contribute to the modulation of the time required for complete drug release, ranging from a few minutes to over one week.

Still in the area of prophylaxis, the team led by Banga at Mercer University and CONRAD, USA, propose a new transdermal delivery system for tenofovir alafenamide, a nucleotide reverse transcriptase inhibitor [25]. The silicone-based patch was shown to be able to provide in vitro sustained drug release that may potentially allow weekly cutaneous applications for the purpose of systemic PrEP. Another exciting alternative for the delivery of tenofovir alafenamide was reported by Johnson et al. at RTI International and PATH, USA [26]. These researchers provide details on the manufacturing and in vitro evaluation of a subcutaneous reservoir-style implant for long-term delivery of the drug. In particular, sustained release was achieved for an impressive period of 180 days, representing an important step towards the development of a putative long-acting product for systemic PrEP or even therapy.

Rohan and colleagues at the University of Pittsburgh, Magee-Womens Research Institute, University of Louisville and International Partnership for Microbicides, USA, contributed an interesting study that further endorses the potential of nanotechnology-based microbicides [27]. In their study, poly(lactic-*co*-glycolic acid)-based nanoparticles were developed as carriers for the co-delivery of griffithsin and dapivirine, two potent candidate microbicide compounds. Studies in vitro showed that the proposed formulation not only featured interesting technological properties (including biphasic drug release), but also allowed a synergistic antiretroviral effect to be obtained. In another paper pertaining to the application of nanotechnology against HIV infection, Grande et al. (University of Calabria, Italy) reviewed the literature for nanocarriers of reverse transcriptase inhibitors [28]. In this interesting article, the authors provide a critical analysis on how nanosystems such as liposomes, niosomes, and solid lipid nanoparticles can help with overcoming technological and pharmacokinetic problems of this important class of antiretroviral drugs.

I hope that researchers involved in the fields of antiretroviral drug delivery and anti-HIV therapy/prophylaxis may find useful and stimulating information here that can be translated into their own ongoing and future work. A final word of appreciation is due to all the contributing authors, anonymous reviewers, and editorial staff at MDPI for making the publication of this Special Issue of *Pharmaceutics* possible.
